# Emergence of Salmonid Alphavirus Genotype 2 in Norway—Molecular Characterization of Viral Strains Circulating in Norway and Scotland

**DOI:** 10.3390/v13081556

**Published:** 2021-08-06

**Authors:** Monika J. Hjortaas, Elena Fringuelli, Adérito L. Monjane, Aase B. Mikalsen, Christine M. Jonassen, Paul Savage, Hilde Sindre

**Affiliations:** 1 Norwegian Veterinary Institute, 1433 Ås, Norway; aderito-luis.monjane@vetinst.no; 2 Veterinary Sciences Division, Agri-Food and Biosciences Institute of Northern Ireland, Stormont, Belfast BT4 3SD, UK; Elena.Fringuelli@afbini.gov.uk (E.F.); Paul.Savage@afbini.gov.uk (P.S.); 3 Department of Paraclinical Sciences, Faculty of Veterinary Medicine, Norwegian University of Life Sciences, 1432 Ås, Norway; aase.mikalsen@nmbu.no; 4 Center for Laboratory Medicine, Østfold Hospital Trust, 1714 Grålum, Norway; Christine.Monceyron.Jonassen@so-hf.no

**Keywords:** salmonid alphavirus, genotypes, SAV2, aquaculture, Norway, Scotland, phylogeography, molecular epidemiology, pancreas disease

## Abstract

Pancreas disease (PD) and sleeping disease (SD), caused by an alphavirus, are endemic in European salmonid aquaculture, causing significant mortality, reduced growth and poor flesh quality. In 2010, a new variant of salmonid alphavirus emerged in Norway, marine salmonid alphavirus genotype 2 (SAV2). As this genotype is highly prevalent in Scotland, transmission through well boat traffic was hypothesized as one possible source of infection. In this study, we performed full-length genome sequencing of SAV2 sampled between 2006 and 2012 in Norway and Scotland, and present the first comprehensive full-length characterization of Norwegian marine SAV2 strains. We analyze their relationship with selected Scottish SAV2 strains and explore the genetic diversity of SAV. Our results show that all Norwegian marine SAV2 share a recent last common ancestor with marine SAV2 circulating in Scotland and a higher level of genomic diversity among the Scottish marine SAV2 strains compared to strains from Norway. These findings support the hypothesis of a single introduction of SAV2 to Norway sometime from 2006–2010, followed by horizontal spread along the coast.

## 1. Introduction

Pancreas disease (PD) and sleeping disease (SD) affect Atlantic salmon (*Salmo salar* L) and rainbow trout (*Oncorhynchus mykiss W*), respectively. The diseases lead to considerable financial losses and affect animal welfare in the aquaculture industry in Northern Europe [1,2]. In Norway, PD was initially found in an endemic region limited to the central western coast in the 1980s, from which it spread northwards and southwards from 2003–2004, resulting in a large endemic region covering almost the entire mid-/southwestern coast [3]. PD and SD are similar diseases in that both are characterized by clinical signs including necrosis and loss of exocrine pancreatic tissue, as well as characteristic histopathological changes in heart and skeletal muscle [1]. The outcome is growth retardation due to loss of appetite, a lethargic or “sleeping” behavior and eventually increased mortality. Fish that survive the disease may subsequently undermine a farm’s production cycle due to reduced fillet quality.

The etiological agents of PD and SD are, respectively, salmon pancreas disease virus (SPDV) and sleeping disease virus (SDV) in the genus *Alphavirus*, within the family *Togaviridae* [4,5]. The viruses are spherical, membrane-enveloped viruses with a single-stranded positive sense RNA genome of approximately 12 kb. Due to similarities between the viruses, the name salmonid alphavirus (SAV) has been suggested to describe both genotypes.

Based on the partial nucleotide sequences of SAV E2 and non-structural protein 3 (nsp3), SAV strains have been classified into six genotypes (SAV1–6) [6,7]. The distribution of these genotypes is, to a certain degree, geographically structured with SAV2 and 3 found in Norway [8,9], SAV1, 2, 4, 5 in Scotland and SAV 1, 2, 5, 6 in Ireland [10]. There is evidence for mixed genotype infection in salmon at the farm level and within genotype strain diversity within the individual fish [11]. Besides infecting Atlantic salmon and rainbow trout, an increasing number of studies have demonstrated SAV infections in non-salmonid fish and it is suggested that these may represent wild reservoirs of SAV. The non-salmonid fish species include flatfish [11,12,13] and Ballan wrasse [14,15], the latter being the source of a newly identified SAV7 genotype [14]. Although each of these various genotypes have mostly been identified in individual fish, sequence analyses of SAV derived from a number of wild flatfish [11] have shown the presence of two genotypes within a single fish, indicating that mixed infections with various SAV genotypes can occur. Clinical signs of PD or SD in wild fish infected with SAV have so far not been observed.

SAV2 is associated with outbreaks of SD in rainbow trout (*Oncorhynchus mykiss*) reared in mid-Norway, and marine SAV2 has subsequently spread from Møre and freshwater facilities. Following its first descriptions in France [16], SD has subsequently been observed in several European countries, including Croatia, Germany, Ireland, Italy, Poland, Spain, Switzerland and the United Kingdom [7]. Moreover, SAV2 causes PD in marine farming of Atlantic salmon in Scotland and Norway [6,10,17]. In Scotland, marine SAV2 strains are extensively prevalent, particularly in Shetland and Orkney, where the circulation of the virus dates back to at least 2006 [10]. Introduction of marine SAV2 to Norwegian aquaculture presumably occurred sometime before 2010 [17], following the first detections in Romsdal County southwards to the northern part of Vestland County and northwards to Trøndelag [3]. The prevalence of SAV2 in both freshwater systems and SAV2 in saltwater is reflected in two recognized genetic subgroups, the so-called ‘fresh water’ (FW) and ‘marine’ strains of SAV2 [10,17]. Viral sequences representing both FW SAV2 and marine SAV2 have been detected in wild-caught common dab (*Limanda limanda*) in Scotland [11,12]. Although pathology and mortality, typically associated with SAV infection, have not yet been demonstrated in non-salmonids, the impact of these seemingly benign infections on the transmission dynamics of FW and marine SAV2 among farmed salmonids is nevertheless of epidemiological relevance. In this regard, the detection of six SAV2 strains sampled from Atlantic salmon in saltwater facilities, yet showing 100% nucleotide identity with several FW SAV2 strains in rainbow trout [10], highlights the potential of virus transmission between fish species (in the freshwater and marine environments), due to ineffective biosecurity measures, anthropogenic processes or introductions from the environment.

Improved characterization of pathogens and deeper insight into the diversity of viral genomes have shed light on the origin, transmission dynamics and evolution of disease. This knowledge has contributed to the development of precise diagnostic techniques and optimized the impact of disease control measures [18,19,20]. Before the emergence of marine SAV2 in Norwegian aquaculture in 2010, all cases of PD were exclusively associated with the SAV3 genotype [8,9,17,21,22]. However, the introduction of marine SAV2 increased the need for routine genetic characterization of all SAV outbreaks at the genotype level, and led to modifications in the control measures to be implemented against PD in Norway [3,17]. In 2014, analysis of a large number of SAV3 genomes showed that this particular genotype was introduced to Norwegian aquaculture as a single event from an unknown source of infection [23]. A more recent analysis of the genetic diversity of SAV3 in selected areas of Norway demonstrated repeated seeding events in Vestland County [24], through a process that hypothetically may have contributed to the country-wide spread of SAV3 since the first introduction.

Although increasing in number [11,24,25,26], full-length or near full-length SAV sequence data represent only a minor proportion of the total publicly available sequence data for SAV. Among the approximately seventy publicly available full-length SAV genomes, only twelve represent SAV2, thirty-five SAV3 and fourteen SAV1. The paucity of full-length SAV2 genomes explains, in part, why most sequence-based analyses of SAV2 have to date been performed using sequence data from relatively short fragments covering partial E2 or nsp3 genes (representing approximately 3% of the full genome length), consequently limiting the possibility to make more robust phylogenetic inferences, or to describe the epidemiological and evolutionary dynamics of SAV2.

In this study, we performed full-length genome sequencing of SAV2 sampled between 2002 and 2012 in Norway and Scotland. Using these and publicly available sequences of SAV allowed us to generate a dataset with greater genomic diversity, which we used to explore epidemiological inferences regarding the introduction of marine SAV2 into Norwegian aquaculture, and to further describe the genetic diversity within the SAV2 genotype. We observed a higher level of genomic diversity among the Scottish marine SAV2 strains compared to Norwegian strains. All the Norwegian marine SAV2 shared a recent common ancestor, supporting the hypothesis of a single introduction of SAV2 to Norway sometime from 2006–2010 from Scotland, followed by horizontal spread along the coast.

## 2. Materials and Methods

### 2.1. Selection of the Viral Strains

The criteria used to select virus strains for sequencing were geography and time of detection and included the years 2002–2012. In total, twelve SAV2 strains, six from Norway and six from Scotland, were included (Table 1). The Norwegian subset included strains from the putative introduction area of marine SAV2 in Møre and Romsdal County, along with strains from the counties of Trøndelag and Troms sampled in the year of estimated introduction and the two following years. Scottish strains were sampled one, four and eight years before the first description of PD caused by marine SAV2 in 2010 in Norway. To reflect the diversity within the SAV2 genotype and balance our dataset, two Scottish SAV2 strains, previously described as FW strains [6,10]—one collected from rainbow trout in fresh water and one from Atlantic salmon reared in a sea facility—were also included.

### 2.2. Origin of Norwegian Strains

**Figure 1:**
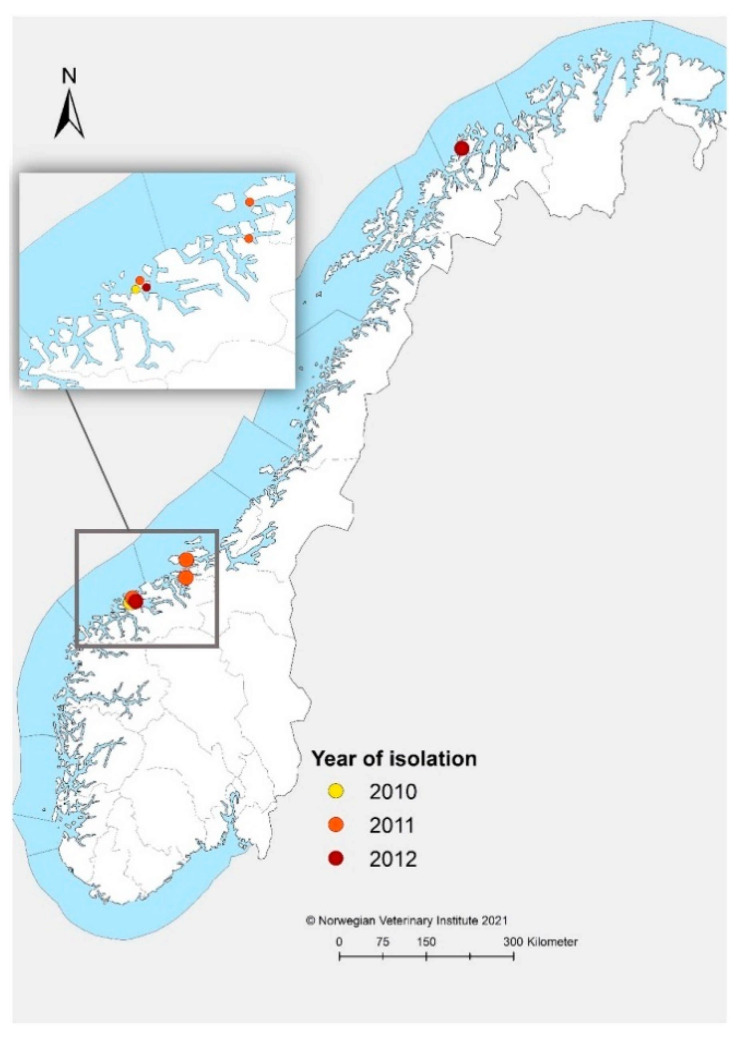
Map showing the geographical location of the six Norwegian sites included in this study. The colored dots represent year of SAV detection at each site. The enlarged window represents farms in Møre and Romsdal County and Trøndelag, while the farm in the northern part of the country is situated in Troms.

The Norwegian strains included five virus strains obtained by propagation in Chinook salmon embryo cells (CHSE-214) in two passages as previously described [27], and one heart tissue sample (MR-R1-2010) on RNA *later*^®^ (Thermo Fisher Scientific, Waltham, MA, USA). The sample material originated from submissions to the Norwegian Veterinary Institute (NVI) between June 2010 and June 2012, and represents the first known cases of infections with marine SAV2 (Figure 1) [8,17] The original samples were screened for SAV by RT-PCR and the genotype was confirmed by sequencing of a portion of the E2 gene as previously described [17].

### 2.3. Origin of Scottish Strains

**Figure 2:**
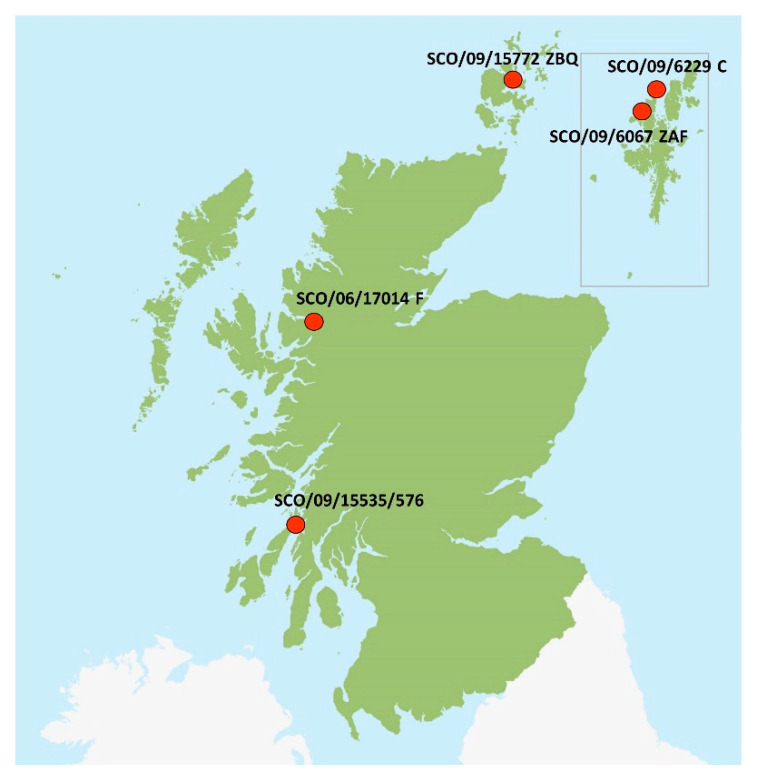
Map showing the geographical location of five of the six Scottish sites included in this study. Information regarding the geographic origin of isolate SCO/09/18948/12225 is not available.

Scottish strains were selected based on the phylogenetic analysis performed by Graham et al. [10]. In that analysis, different monophyletic branches made of identical sequences were identified within the genotype 2. Each one of the six strains is representative of a different branch. In addition, SCO/09/18948/12225 and SCO/09/15535/576, although from the same branch, were selected as they are from rainbow trout and Atlantic salmon, respectively. Moreover, two strains originating from Shetland (SCO/09/6067 ZAF and SCO/09/6229C) represented an area from which Norwegian well boats, based in Møre and Romsdal, were used for transport of smolt in Scotland during 2009 and 2010 (Figure 2).

### 2.4. Sample Preparation, PCR Amplification of Viral Genome and Sequencing

To amplify the full SAV genome, fifteen primer pairs were designed using the Oligo Primer Analysis software (Molecular Biology Insights, Inc. Table S1 (Supplementary Material) provides an overview of the position of primers along the genome of sleeping disease virus (GenBank: AJ316246).

For each sample, RNA was extracted as previously described [22] and cDNA synthesis was performed using Superscript III reverse transcriptase in combination with random hexamers (both Invitrogen), following the protocol recommended by the manufacturer. The cDNA was used as a template in fifteen separate PCR reactions to generate overlapping PCR products of the SAV genome; in total 11,700 nt. PCR was performed using high-fidelity AccuPrime *Pfx* DNA polymerase (Invitrogen) with primers as shown in Table S1 (Supplementary Material). The reactions were run in a total volume of 25 µL with 3 µL cDNA and 7.5 pmol of each primer using the following thermal conditions: 95 °C/2 min, followed by 40 cycles of 95 °C/15 s, 55 °C/30 s and 68 °C/2 min. The correct size of PCR products was confirmed by either electrophoresis on a 1.5% agarose gel or by fragment analysis using a 2100 Bioanalyzer (Agilent Technologies). PCR products were purified using ExoSAP-IT (Invitrogen) following the manufacturer’s protocol. Sanger dideoxy sequencing of the PCR products was conducted using amplification primers and internal forward and reverse primers designed for each fragment with an ABI Prism Big Dye Terminator Cycle sequencing kit on the ABI Genetic Analyzer at the NVI. Sequences were assembled using the Sequencher 4.5 software (Gene Codes Corporation, Ann Arbor, MI, USA).

### 2.5. Data Analysis

Additional genomic sequences of SAV were downloaded from GenBank and from Supplementary Material, Dataset S1 presented by Gallagher et al. [11]. These were subsequently aligned using the MUSCLE algorithm and manually curated in MEGA version 10.1 [28]. Two alignment datasets were generated based on near full genome (11612 nt long) and nsp3 (1760 nt). Using each dataset, the best fitting model of nucleotide substitution (GTR + G4) was inferred using PhyML3 [29] as implemented in RDP4 version 4 (software for recombination analysis; University of Cape Town; South Africa; 2015) [30], and maximum likelihood (ML) trees were estimated using PhML3, which were used to calculate pairwise genetic identity values between sequences within the full-genome alignment. MEGA version 10.1 [28] was used to establish the genomic diversity within each coding sequence of SAV.

## 3. Results

In this study, we sequenced twelve new SAV2 genomes (eight from 8575–11,766 nt and four from 990–1912 nt) to add to the database of SAV genome sequences. Eleven of these isolates were grown in cell culture prior to sequencing, whereas one was sequenced directly from a tissue field sample. All sequenced viruses were obtained from either Norway or Scotland. The Scottish strains were collected in 2002, 2006 or 2009, which equate to, respectively, eight, four or one year before the first description of PD caused by marine SAV2 in 2010 in Norway. The Norwegian strains were sampled from 2010–2012, and included strains from the putative introduction area of marine SAV2 in Møre and Romsdal, along with strains from PD outbreaks resulting from the initial northward spread of this genotype to the counties of Trøndelag and Troms. The sequencing generated near full-length genomes of approximately 11,600 nt in length, excluding the 5′ and 3′ termini, from five Norwegian strains and three Scottish strains. PCR amplification sensitivity varied for one Norwegian and three Scottish strain samples and overlapping PCR products covering the full-length genome were not achieved. This resulted in partial genomic sequences representing these strains varying in size from 990–8575 nt (Table 1).

### 3.1. Phylogeny of SAV

**Figure 3:**
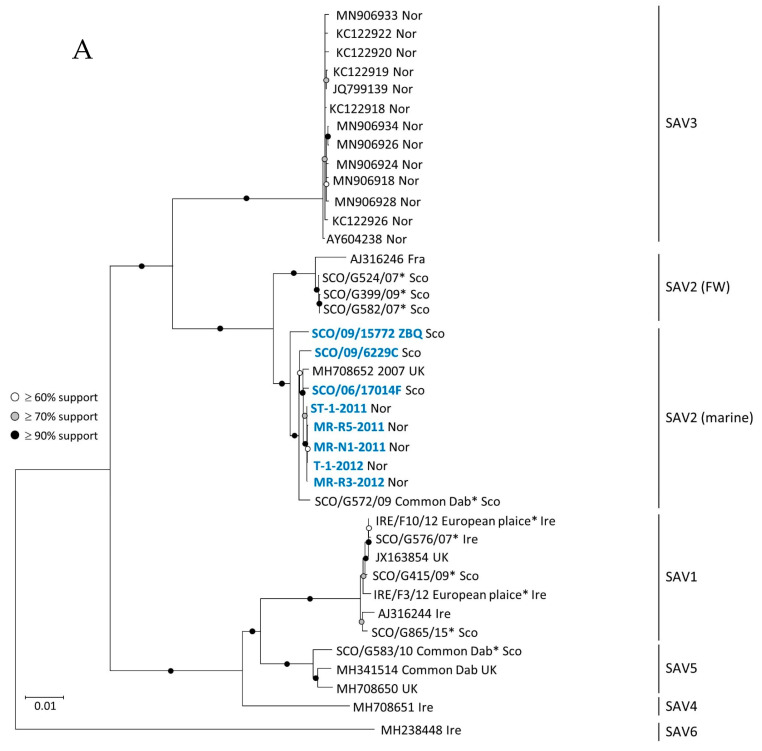

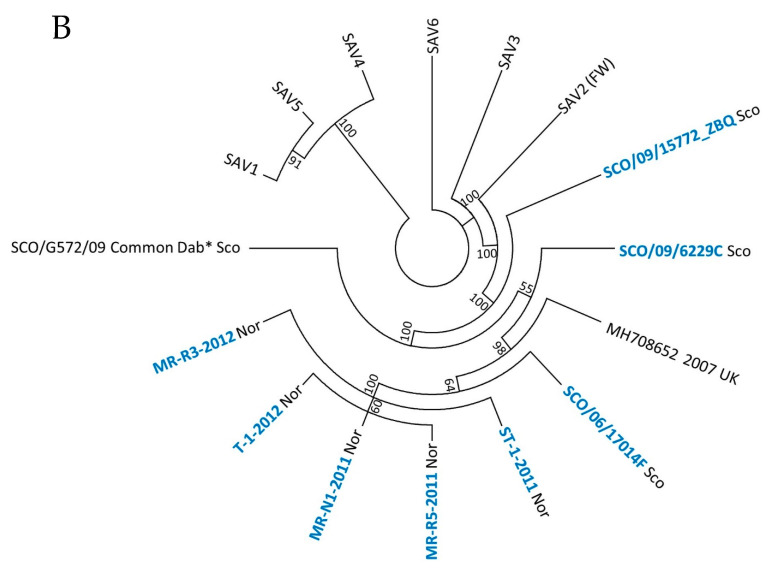
Maximum likelihood trees showing the relationship between strains of SAV1–6. (**A**) The tree includes representative sequences from each genotype. (**B**) Graphical simplification of (**A**) with all sequences in the clades for SAV1, SAV2 (FW), SAV3, SAV4, SAV5 and SAV6 collapsed to expand the size of the marine SAV2 clade. The tree was inferred using a 11,612 nt nucleotide alignment of SAV genomes including eight new SAV2 generated in the present study (in blue), SAV genomes obtained from GenBank (referred to by the accession number) and Supplementary Material, Dataset S1 presented by Gallagher et al. [11], marked with (*). Country/geographic area of origin is marked by a suffix at the end of each sequence. Isolate with accession number MH708652 originates from Scotland [25].

**Figure 4:**
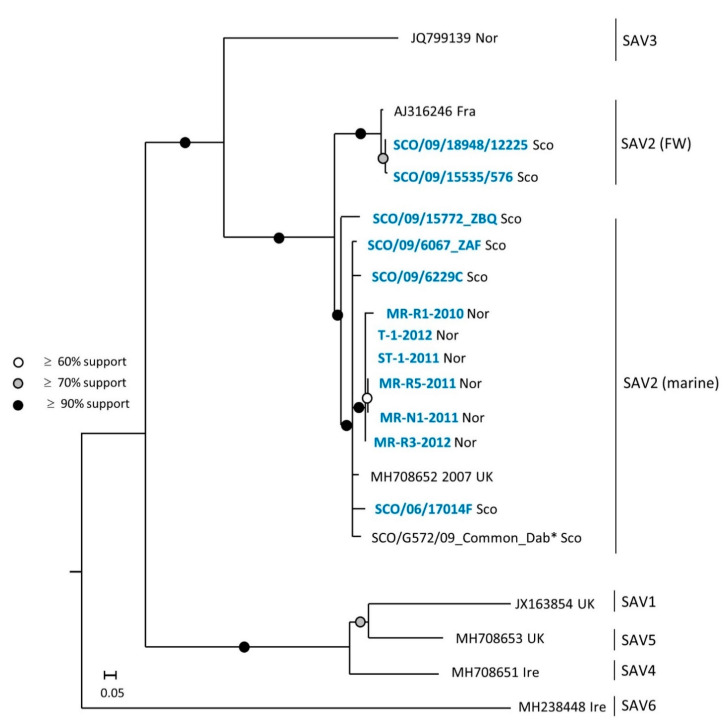
Maximum likelihood trees of a 1760 nt fragment covering the nsp3 gene to identify the relationship between Norwegian and Scottish SAV2. Twelve new SAV2 nsp3 generated in the present study (in blue) are included together with SAV1–6 nsp3 sequences obtained from GenBank (referred to by the accession number) and SAV2 nsp3 sequences from common dab from Supplementary Material, Dataset S1 presented by Gallagher et al. [11], marked with (*). Country/geographic area of origin is marked by a suffix at the end of each sequence. To improve the graphical visualization, only selected sequences from the FW SAV2 subgroup are shown. The analysis was also performed including the three FW SAV2 sequences from the full genome dataset in Figure 3 with identical results.

Two maximum likelihood trees were inferred using two different datasets: (i) a full-length genome (11,612 nt) dataset, shown in Figure 3, (ii) and an nsp3 dataset (1760 nt), shown in Figure 4. The nsp3 dataset includes sequence data from the nsp3 region of all the viruses included in the full genome dataset, but also nsp3 sequence data from four additional strains from which only partial genome sequences were generated in this study. These four additional strains are: FW SAV2 found in Atlantic salmon at a marine site (SCO/09/15535/576), FW SAV2 detected in a freshwater production site of rainbow trout (SCO/09/18948/12225), a marine strain (SCO/09/6067 ZAF) and a marine SAV2 strain (MR-R1-2010) originating from the first detection of SAV2 in Norway [8].

Phylogenetic analyses performed on the full-genome dataset provided maximum statistical support for a monophyletic origin of all SAV2 strains, and they suggest that SAV2 consists of two distinct clades, namely the FW SAV2 and marine SAV2, as described previously [6,10]. The clustering of Norwegian and Scottish sequences within the marine subgroup of SAV2 was supported by bootstrap values over 90%. Strain SCO/06/17014F detected in 2006 in northwest Scotland shares the most recent common ancestor with Norwegian strains of SAV2 (Figure 3).

The same relationship between Norwegian and Scottish SAV2 was observed when analyzing the 1760 nt nsp3 gene dataset (Figure 4). Moreover, the pairwise comparison data showed that all six Norwegian SAV2 viruses (marked with blue font in Figure 4) generated in this study were 98–99% identical at the nucleotide level. The virus strain MR-R1-2010, which represents the first detection of the marine SAV2 in Norway, was grouped within a cluster of four marine SAV2 viruses sampled in Scotland within four years of the putative introduction of SAV2 to Norway. The two Scottish sequences, SCO/09/15535/576 and SCO/09/18948/12225, were nearly identical and clustered within the FW SAV2 subgroup, as expected. The strain SCO/G572/09 from common dab was more distantly related to the Norwegian marine SAV2, based on the phylogenetic analysis of the full-genome dataset. This was less apparent in the nsp3 tree where clustering of the sequence from common dab indicated a closer relation to the strains derived from salmonids.

### 3.2. Genomic Diversity of SAV

**Figure 5:**
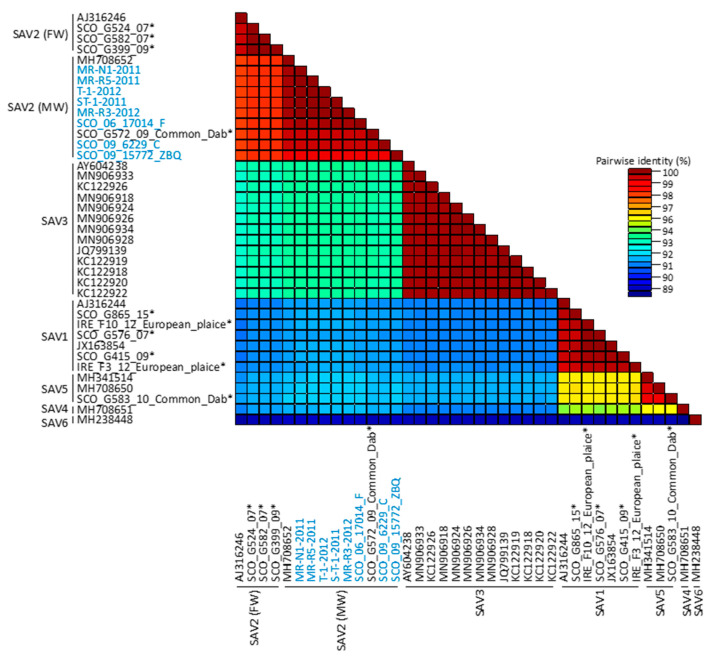
Pairwise identity matrix representing genomic diversity across and within SAV genotypes 1–6 performed on sequences within the full-genome dataset shown in Figure 3. Included are SAV2 genomes generated in the present study (in blue) and reference SAV genomes for each genotype obtained from GenBank (referred to by the accession number) and SAV genomes published in Supplementary Material, Dataset S1 presented by Gallagher et al. [11], marked with (*). SAV2 (MW) = marine SAV2.

Analysis of genomic diversity within the full-genome dataset is presented in Figure 5. As previously reported, the divergence across the genotypes tended to be higher when compared to the divergence found within any single genotype [6]. For example, we observed the highest nucleotide divergence between SAV6 and the remaining subtypes, and the lowest between SAV1 and SAV5. Within the genotypes, genetic diversity is generally low, on average between 1 and 2%.

Overall, the SAV2 sequences generated in this study were highly conserved, with an average pairwise genomic nucleotide and encoded amino acid identity of 98.7% and 99.5%, respectively. Among the Scottish strains, genomic nucleotide diversity of 1.2% was observed, while Norwegian SAV2 viruses were almost identical with only 0.05% diversity, corresponding to six nucleotide substitutions (of which only one in the *nsp4* gene was non-synonymous) along the 11.7 kb genome, a pattern consistent with recent the introduction and horizontal transmission of SAV2 in Norway. Analysis of coding sequences of SAV2 generated in this study showed that regions covering the nsp3 and E2 genes were the most diverse with, respectively, 2.2% and 1.9% nucleotide substitutions each. Therefore, subsequent analyses of intra-genotype diversity were performed on those two genes, allowing us to include publicly available sequence data from the FW variants of SAV2 for which only partial genome sequences are available. This addition increased nucleotide diversity for both genes to 4.1% and 4%, respectively

### 3.3. Characteristic of Genomic Deletions in FW SAV2 Strains and SAV3

**Figure 6:**
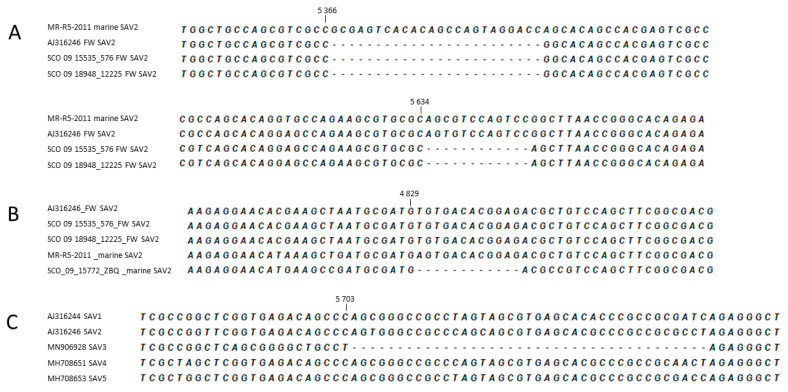
Graphical presentation of deletion patterns throughout the nsp3 gene of SAV2 and SAV3 strains. Positions of the deletions are relative to the FW SAV2 strain s49p (AJ316246). (**A**) The 12 nt and 24 nt deletions observed among the FW SAV2 variants, (**B**) the 12 nt deletion close to the 5′ end of marine SAV2 strain SCO/09/15772 ZBQ from Orkney and (**C**) the 39 nt deletion within SAV3 genotype.

Alignments of the FW and marine SAV2 variants in the study revealed deletions in the consensus sequences of the genomes of the FW strains compared to marine strains at two positions in the nsp3 gene (Figure 6A). The deletions were both located towards the 3′ end of the gene with sizes of 24 nt and 12 nt, respectively, and did not change the reading frame. The 24 nt deletion was also present in all the five FW SAV2 genomes published previously, with exception of the variant originating from wild dab. Moreover, all the remaining SAV genotypes presented a full-length sequence at that particular position. The 12 nt deletion, also described in a previous study [6], seems to be exclusive to FW strains generated in this study. Neither of these two deletions were present within any available marine SAV2 genomes; however, another 12 nt deletion close to the 5′ end of the nsp3 gene was observed in the strain SCO/09/15772 ZBQ from Orkney (Figure 6B). Screening for indels across the six SAV subtypes in the dataset used to generate Figure 5 revealed the presence of deletions that are 3 nt and 39 nt long in the nsp3 gene of the 13 strains included in the SAV3 group. The 39 nt deletion (nt 5698–5737, relative to reference strain s49p; AJ36246) was not present among the remaining SAV genotypes (Figure 6C). Additionally, an alignment including all published sequences of the SAV3 nsp3 gene revealed that this deletion was present in all SAV3 genomes (*n* = 30), including both the oldest available strain from 1997 and more recent ones from 2019. The 3 nt deletion (nt 5602–5604) was observed in sequences of both SAV3 and SAV1 genotypes.

## 4. Discussion

In this paper, we present the first comprehensive full-length characterization of Norwegian marine SAV2 strains, analyze their relationship with selected Scottish SAV2 strains and explore the genetic diversity of SAV. We show that all Norwegian marine SAV2 share a recent last common ancestor with circulating Scottish marine SAV2 and observe a higher level of genomic diversity among the Scottish marine SAV2 strains compared to strains form Norway. Our results support the hypothesis of a single introduction of SAV2 to Norway sometime from 2006–2010 (note that our phylogenetic analysis suggests that the SAV2 isolate most closely related to the Norwegian one detected in 2010 already existed in Scotland in 2006), followed by horizontal spread along the coast. This picture is compatible with the theory of transmission from Scottish aquaculture to Norway as one possible source of infection, facilitated by the use of Norwegian well boats in Scotland [8].

Investigations of the distribution of SAV in Atlantic salmon farms in Scotland revealed the presence of subtypes 1, 2, 4 and 5 in infected fish, and indicated that particular genotypes dominate specific geographical regions [10]. For example, in the Western Isles, subtypes 4 and 5 were the dominant ones, while marine SAV2 appears to be widespread in the Shetland Islands and Orkney. Four Scottish strains of marine SAV2 collected within three years of the putative introduction of SAV2 to Norway were included in our study. Although careful sample selection criteria were applied in this study to ensure that the strains sequenced originated from geographical areas with high prevalence of SAV2, the four strains selected nevertheless represent only a minor portion of a large pool of SAV2 isolates obtained from marine farms distributed across a wide geographical area in Scotland. Due to this, any maximum likelihood-based inferences made as to the exact site in Scotland that served as the source of infection in Norway would naturally be limited to the geographical areas from which the four sequenced strains were obtained. In spite of this caveat, we were at the very least able to demonstrate that the Norwegian marine SAV2 isolates form a monophyletic clade with Scottish marine SAV2 isolates in general, thus strengthening the hypothesis that all Norwegian outbreaks of PD caused by SAV2 share a common source of infection in Scotland.

Both the full-genome dataset and that of the significantly shorter nsp3 gene support a hypothesis of a single introduction of marine SAV2 to Norway from Scotland. Although these datasets do not provide sufficient resolution to confidently pinpoint where exactly in Scotland the infection originated, it is worthwhile noting, however, that among all the Scottish SAV2 strains used in the full-genome dataset, the SCO/06/17014F strain detected in northwest Scotland, sampled four years before first detection of SAV2 in Norway, is the one that shares the most recent common ancestor with the Norwegian SAV2 viruses. This implies that (keeping in mind the caveat stated earlier) marine SAV2 infections originated in Scotland, likely northwest Scotland.

The low genetic diversity among Norwegian marine SAV2 strains collected close to the time of the putative introduction to Norwegian aquaculture, shortly before or during 2010, is consistent with a single transmission event of SAV2 followed by horizontal spread to other salmon production sites along the Norwegian coast. This observation is further supported by differences in the estimated genomic diversity for the Scottish and Norwegian marine SAV2 strains. In this study, genomic diversity within the Scottish marine SAV2 strains (sampled from 2002–2009) was 1.2%, but only 0.05% among the Norwegian SAV2 strains (sampled from 2010–2012). The larger diversity within the Scottish marine SAV2 population indicates that the region of Scotland covered in our study is likely a site of a SAV population with large marine SAV2 diversity, from which a single transmission event to Norway may subsequently have occurred.

How SAV2 was introduced to Norwegian aquaculture is still unknown. However, well boats harboring at the putative introduction area in mid-Norway have been involved in transport of smolt between farms in the Shetland Islands and Orkney, two areas with a high prevalence of marine SAV2 [10]. Although it is not known to what extent there was an exchange of live fish and/or well boat traffic between these areas and northwest Scotland during the relevant time period, it is conceivable that anthropogenic transport activity may have played a role in the introduction of SAV2 to Norway, a possibility that is credible given that transportation of fish by ship has been linked to the spread of infectious salmon anemia virus in Scottish aquaculture [31].

However, given that a wild reservoir of SAV is reported to likely be located in or around the North Sea [23], a counter argument could be made that anthropogenic activity is not the cause of SAV2 introduction to Norwegian aquaculture. Marine SAV2 has been found in wild common dab in Scotland [12] and introduction of SAV2 to Norway from a wild fish reservoir, e.g., through direct contact between species or illegal feeding of cultured salmon with raw wild fish, cannot be excluded. It is, however, difficult to validate this possibility because extensive screening for SAV in wild fish in Norway is still lacking. Based on the phylogenetic analysis of the full-genome dataset, the strain SCO/G572/09 from common dab was more distantly related to Norwegian marine SAV2. However, this was less apparent in the nsp3 tree where clustering of the sequences from common dab and salmonids suggests a closer relation. The slightly different story told by those two datasets may reflect different evolutionary rates and pressures exerted on genes encoding the non-structural proteins and structural proteins, as well as host-specific features, including host adaptation. Given these nuances, the analysis based on the full dataset is possibly more robust and informative since it incorporates more evolutionary information from both the structural and non-structural genes, as opposed to only from the nsp3 gene. Still, since the phylogenetic analyses show a close relationship between the Scottish and Norwegian salmon SAV2 isolates and the isolates sharing the closest most recent common ancestor are those sampled from salmon, it is more plausible that SAV2 in Norway resulted from transmission from farmed salmon. To validate this hypothesis, SAV2 sequence data from a larger population of wild flatfish should be included in future analyses.

Several deletions in the consensus sequence of the nsp3 gene were observed close to the 3′ end, both within SAV2 and when compared across genotypes of SAV. Similarly, a deletion was found in SAV3 nsp3, compared to the remaining genotypes. Although of varying lengths, neither of the deletions disrupted the open reading frame, suggesting the production of viable, infective virus particles. These in-frame deletions may have resulted from adaptations of SAV to different aquatic environments and/or host species and may represent specific features of certain genotypes or subgroups. On the other hand, some of the deletions could result in relatively large changes in the nsp3 protein, which may have influenced its biological properties, possibly resulting in reduced fitness or production of defective virus. For instance, the 24 nt deletion in the FW SAV2 genome would lead to an eight amino acid long deletion, while the 39 nt gap in SAV3 implies a deletion of as many as 13 amino acids. The precise function of the nsp3 protein during infection has been unclear for a long time, but a crucial function of nsp3 as a mediator protein in several virus–host protein–protein interactions has been suggested, particularly through the C-terminus of the protein, characterized as a hypervariable domain (HVD) [32]. Although the HVD of alphaviruses is highly variable between related alphaviruses, the domain contains features linking it to a function as a mediator of multiple host–protein interactions, suggesting involvement in host adaptation processes [32,33,34]. Studies have shown that although it is essential for alphavirus replication, the HVD tolerates deletions and insertions [35,36,37], as long as the critical single residues remain conserved [38,39]. These features may be beneficial for optimal virus replication in diverse host cells. All deletions described in our study relate to the part of nsp3 in alphaviruses described as an HVD and, for SAV, described as less conserved [5]. The 24 nt deletion described in FW SAV2 in this study has been previously reported by Weston and co-workers [5] and seems to be a genotype-specific feature of FW SAV2 in salmonids. This may be a result of evolutionary adaptation to the host, as this deletion was not present in FW SAV2 originating from wild dab. The presence of a 39 nt deletion specific to SAV3, also found in the HVD of nsp3, supports that genotype- or host-specific deletions seem to be a characteristic for SAV.

The 12 nt deletion in FW SAV2 described here has also been reported previously in one FW SAV2 isolate from rainbow trout, but was not found to be present in the nsp3 sequence of any other FW SAV2 included in that study [6]. Since it was only found as a feature among a selection of FW SAV2 strains, which in our study also included a FW SAV2 strain found in salmon in a marine production phase, it may not be a result of evolutionary adaptation to the host, but could instead be connected to virulence. Data for a detailed comparison of clinical severity of the disease outbreaks related to the described FW SAV2, with or without this deletion, are lacking and should be a focus in future studies.

Although Sanger sequencing has certain limitations compared to NGS, including the amount of data produced, speed of analysis and sequencing [40], it performs well in applications aiming towards revealing of the dominant viral strain in a sample. In our case, the analysis of Sanger sequenced data sufficed to point to a single introduction of marine SAV2 in Norway sometime from 2006–2010, and that this founder population shared a recent last common ancestor with marine SAV2 strains circulating in Scotland. Given the rapid evolutionary rate of RNA viruses, and the fact that some of the genetic diversity in host populations may arise out of amino acid deletions, this study further shows that the evolution of SAV strains warrants further high-throughput and deep-sequencing studies.

## Figures and Tables

**Table 1:** Geographical origin and date of sampling of the SAV2 strains. All viruses originated from marine production sites stocked with Atlantic salmon, except SCO/09/18948/12225, which originated from rainbow trout at a freshwater facility. Sequences from the strain MR-R1-2010 were obtained directly from the heart tissue; remaining viruses were cell culture isolates. The sequence length of each strain achieved in this study is also given. * Dataset S1 Supplementary Material.

Strain	Geographic Origin	Date of Sampling	Host	Site	Acc No	Genotype	Sequence Length (nt)
Norwegian							
MR-R1-2010	Møre and Romsdal	June 2010	A. salmon	Marine	*	Marine SAV2	2252 and 990
MR-R5-2011	Møre and Romsdal	Oct 2011	A. salmon	Marine	MZ395641	Marine SAV2	11,766
MR-N1-2011	Møre and Romsdal	Dec 2011	A. salmon	Marine	MZ395642	Marine SAV2	11,658
ST-1-2011	Trøndelag	Dec 2011	A. salmon	Marine	MZ395643	Marine SAV2	11,719
T-1-2012	Troms	Jan 2012	A. salmon	Marine	MZ395644	Marine SAV2	11,753
MR-R3-2012	Møre and Romsdal	June 2012	A. salmon	Marine	MZ395645	Marine SAV2	11,751
Scottish							
SCO/09/6067 ZAF	West Shetland Island	2009	A. salmon	Marine	MZ395646	Marine SAV2	8575
SCO/09/6229C	North Shetland Island	2009	A. salmon	Marine	MZ395647	Marine SAV2	11,594
SCO/09/15772 ZBQ	Orkney	2009	A. salmon	Marine	MZ395648	Marine SAV2	11,409
SCO/06/17014F	Northwest Scotland	2006	A. salmon	Marine	MZ395649	Marine SAV2	11,595
SCO/09/15535/576	Argyll and Bute	2009	A. salmon	Marine	MZ395650	FW SAV2	1770
SCO/09/18948/12225	Unknown	2002	R. trout	Fresh water	MZ395651	FW SAV2	1912

## Data Availability

Salmonid alphavirus sequences obtained in this study have been deposited in the National Center for Biotechnology Information (NCBI) database with accession numbers MZ395641–MZ395651.

## Supplementary Materials

The following are available online at https://www.mdpi.com/article/10.3390/v13081556/s1, Dataset S1: Genomic sequences of the marine SAV2 strain, MR-R1-2010, originating from the first detection of SAV2 in Norway in 2010, Table S1: Details of PCR primers used to generate overlapping products for sequencing.
