# Supplementary material for: Emergence of Salmonid Alphavirus Genotype 2 in Norway—Molecular Characterization of Viral Strains Circulating in Norway and Scotland
Source: Viruses. 2021 Aug 6;13(8):1556. doi: 10.3390/v13081556 (PMC8402823; doi:10.3390/v13081556)
Supplement: Supplementary file 1 [file viruses-13-01556-s001.zip › viruses-1288045-supplementary.pdf]

viruses

MDPI

Supplementary Material

Emergence of Salmonid Alphavirus Genotype 2 in Norway - Molecular Characterization of Viral Strains Circulating in Norway and Scotland

M J Hjortaas, E Fringuelli, A L Monjane, Christine M Jonassen, Aase B Mikalsen, P Savage and H Sindre

Dataset S1. Genomic sequences of the marine SAV2 strain, MR-R1-2010, originating from the first detection of SAV2 in Norway in 2010. Sequences are labelled in the following format:
'Strain name'/'gene'
>MR-R1-2010/nsp2_nsp3
TGAAGACTACGAACCCGTGCAAGAAGCCGGTACTGATTGATACAACCGGG
TCTACCAAGCCTGACAAAGAAGCGTTGGTATTGACGTGCTTCCGCGGGTG
GGTTAAAGATTTAAAAATTCTCTACCCTCACAACGAGCTCATGACTGCGG
CTGCCTCACAGGGTCTGACTCGTGAAAAAGTGTACGCCGTTCGTTGCCGC
GTCACGTCGAACCCACTCTACGAGCCGACTTCTGAGCACATTACTGTCCT
TTTGACGCGCACCAACGACGAACTGGTCTGGAAGACACTGCCAAACGATC
CGCTGATCCCTATACTCTCCAAGCCCCCGAAAGGAGACTACTCCGCCACC
ATGGAGGACTGGGAGGATGAGCACAACGGTATCTTGGCGGCTCTCAGAGA
AGCATGTGTCCCACGGATGAACTTCGCGCACGGGAAGCGTAACACCTGTT
GGGCAGTTACAAGCAGCCGGGTGCTGCACGAGGCAGGCGTCCTGATAACG
CCGGAGGACTTCAACCGCATCTTTCCGGCGTTCCGAGAGGACAAACCGCA
CTCGGCTTTGGCAGCCTTGGATGCTGTCGCCGCTCTCGTGTGGGGCCTGG
ATACATCCTCGGGCATCTTGAGTGGAAAAGGCAGTTTCATGCGTCTGGAG
AACAGCCATTGGAGCAATTCCAACAGAGGGTATGAGTATGGCCTCAACCT
GGACGCACTGGAAGGCTACGAGATCGCCAACCCACGCATGATCAAAGCGC
TTAAACAACGGCGCGGGCGTGAATGTTACGACATTGAAACGGGCAAGTTG
GTGCCTATGGATCCTGGTAGGGTCCAAGTGCCGATCAACCGGGTTGTGCC
CCACGTGCTGGTGGACACTTCCGCAGCGGCCAAACCAGGTTTTCTGGAAA
ACAGGCTCACGGTTGACAGATGGGACCAAGTGCACAGCTTCAAAACAAGA
GCAGCAGTCAAGTTCGCAGAGCTGACGAAGCGCGTCTCATACAACTCAGT
TCTCGACCTTGGGGCCGCCCCGGGAGGAGTGACCGACTATTGCGTGAAGA
AGGGTAAGACCGTTACATGTGTCTCCGAACAGTGGGACTCTAAGCCGAGA
GGCGCTGTGGTCATTACAGCCGATATCAACGGCCCACTTAACAACCTAGG
CATCTTCGACCTGGTGTTTTGTGACGCAGCCGGACCACGACGCTATCACC
ACTACGCGCAGTGCGAAGACCACGCCGTGCTATTCACATCAGCATGCAAA
CACGGGGTGGAGCGCACAGCCAAGGGCGGGGTATTCATTGTGAAGGCGTA
CGGAATGGCGGACCGCCGAACAGAGCGGGCAGTGGAATGCACAGCGAGGT
ATTTCAAGTCCGTCTCGGTCGAAAAACCAGTCTCCTCCCGCATAACCAAC
GTGGAAGTCTTCTTCAAGTTTTCCGGACGCTGTCGCCCGCATGCTCGTTC
CATTGCACACTTGGGCCCTCAACTGACCGACATCTATGCTCGCACGAGGA
AGGCGTACAAAATGCTGGCGAGAGGAAGTGTTGCTGACAAGGTGAAAGTG
GCAGAGATCCTCAACTCGATGGTGGGAGCCGCGCCGGGTTACAGAGTCCT
CAACAGGAACATCATCACTGCCGAAGAAGAAGTCTTGGTTAATGCCGCCA
ACAGCAACGGCAGACCCGGCGACGGTGTGTGTGGTGCGCTCTACGGCGCG
TTCGGGGACGCTTTCCCCAACGGTGCGATCGGCGCGGAAACGCGGTCCT
GGTCCGAGGACTCGAGGCCACCATCATCCACGCAGCCGGAGCTGACTTCA
GAGAGGTCGATGAAGAAACTGGTGCGCGACAGCTGAGAGCAGCATACCGC
GCGGCGGCTACTTTAGTCACTGCTAACGGTATCACCAGCGCTGCCATCCC

TCTGCTGAGTACACACATCTTTTCCAACGGTCGAAACAGACTGGAACAGT
CCTTCGGCGCATTAGTGGAGGCGTTCGACACGACAGAGTGCGACGTCACC
ATCTACTGCCTGGCCAACAACATGGCCGCGAGGATCCAGCAACTAATCGA
CGATCACGCTCGCGAAGAGTTCGACGAGGAAGTGGTTGTGGAAGAAGAAG
AGGAACATAAAGCTGATGCGATGAGTGACACGGAGACGCTGCCCAGCTTC
GGCGACGAAACGGTGTGGGTGCCCAAACATAGTACTCTGGCCGGAAGACC
AGGATACAGTGCCACTTACGGCGACCGCAGATCTCTTTTGTCGGCACGAA
GTCC

>MR-R1-2010/nsp2_nsp3
CCAAAGAAGCCAATGCCAAACTCATTGAGTACATACGAGGGCAACACCTC
GTTGACGTCCTAAAAAGCTGTCCTGTCAACGACATACCGGTAGGTAGACC
GCCTTCTAGCCTGCCCTGCGGTTGCATTTACGCCATGACCCCGGAACGGG
TCACAGTGCTGAAGCAAAGGCCGCAAGAAGGTTTTGTGGTATGCAGTGCA
TTCAAACTACCGCTCACCAACATCCAAGATGTCACCAAAGTGGAGTGCAC
GGGGAGAGCACCTGCAGAGGAACCTAGACCGGTGCGCTACTTGCAAGAGA
GGCGCCCAGCGCAGGCCGCCGCGAGGCAGCCTAGGCCGGCAACTGTGGCT
GCCAGCGTCGCCGCGAGTCACACAGCCAGTAGGACCAGCACAGCCACGAG
TCGCCGCACACCCGCGCCGGGCTCGGTGCAGGTGCGCCTACTGCCGCCAA
GAGACGGCACGGTATCCCGCAGTTCTCGCACGGGTTCGCAGTCCAGCGTC
ACCTCGTCAGCGGGATCCATAATGCCGGTGCCCCGAAGGGCGCCAGTCGC
GCCAGCGGCATCATTGGCGAGCAGCGTCCACAGCCATAGTGTGCGCAGCG
CCCCTGCCATTCTGAGGGCCGCCAGCACAGGTGCCAGAAGCGTGCGCAGC
GTCCAGTCCGGCTTAACCGGGCACAGAGAGAACGCTGTTAGCGTCGCCAG
CTCGGTGAGACAGCCCAGTGGGCCGCCCAGCAGCGTGAGCACGCCCGCCG
CGCCTAGAGGGCTAACACGGGATCAGTTCGGCGCCGTGAGAGCTAGGGCC
CGCAGGGACCTAGAGTTGGAGGGATCGGAGCATGGCAGCCAGGCCAGTTT
CCGTTCCGGCTCGCTGGTGGTGGGGAGCACCGCTAGTAGCTACAGCCAAC
GTCCTGACGACCAGGACACGGGCTCTGAGCCCTCAGGCCGCGGCGCCGCC
GTGAGGACACGACGCAGAGGGCAACGGGACGGCCCCGGAGGGTAT

Table S1. Details of PCR primers used to generate overlapping products for sequencing.

Primer Name	Position	Primer Sequence, 5’ - 3’	Product Length, nt
16	320	GCAAATGCGCTGAAGACCCT	1346
	1644	GACTTCCTCTTCGTCGTCCTGT	
75	1449	CTCCGCCACATACCTTAACCTG	1648
	3077	CGCCAAGATACCGTTGTGCT	
140	2653	CGTTGCACTCATACGCTTACGG	1222
	3854	ACACCAGGTCGAAGATGCCTA	
119	3303	CCTGGATACATCTTCGGGCAT	961
	4242	CTCTGCCACTTTCACCTTGTC	
36	4092	CTCCCGCATAACCAACGTGGAA	910
	4983	AGCCGCTTCGATTGACGAC	
120	4807	GAGGAACACGAAGCTAATGCGAT	490
	5277	GCACCGGTCTAGGTTCCTCT	
124	5012	CCAAAGAAGCCAATGCCAAACTC	500
	5491	CATTATGGGTCCCGCTGACGA	
150	5270	CACCTGCAGAGGAACCTAGACCG	499
	5747	CCGAACTGTTCCCGTGTTAGCC	
167	5583	CAGCGCCCCTGCCATTCTGAG	471

	6031	ACCTCCGTAGTGTTGTTTGTCTG	
189	5902	GACACGGGCTCTGAACCCTC	1504
	7384	ACACTACTCCGGTTATGACGTT	
237	6539	CCGGTCCTATGGCATCTACTCT	1241
	7760	CGCGCCCAAGTACGATCTCA	
Capsid 1F	7505	GCTTCCTGTTACTCGACACGGT	1354
Capsid 1R	8836	CCAGTAGTTCGGGTCATTCAGGT	
274	8731	TCGCCTGCACCTACAACTCCA	1653
	10363	CCGACCACCCTCTTACCCCTA	
324	9757	TCGCCCCACTGCCATCGATT	1644
	11381	CCCCGTACTCGCAACTGTCC	
92	11293	GTGCCGTCCCCATGTCCAT	581
	11850	ACTCATCCTACTCCCTGTGGGTTC	
